# Prognostic value of fibrosis-5 index combined with C-reactive protein in patients with acute decompensated heart failure

**DOI:** 10.1186/s12872-023-03530-2

**Published:** 2023-10-04

**Authors:** Ziyan Wang, Guannan Li, Rong Huang, Lei Chang, Chenyi Gong, Ke Chen, Lian Wang

**Affiliations:** 1 Department of Cardiology, Nanjing Drum Tower Hospital Clinical College of Jiangsu University, Nanjing, 210008 Jiangsu China; 2 Department of Cardiology, Nanjing Drum Tower Hospital, Affiliated Hospital of Medical School, Nanjing University, Nanjing, 210008 Jiangsu China; 3 Department of Cardiology, Nanjing Drum Tower Hospital Clinical College of Nanjing Medical University, Nanjing, 210008 Jiangsu China

**Keywords:** Fibrosis-5 index, C-reactive protein, Acute decompensated heart failure, Major adverse cardiac and cerebral events

## Abstract

**Background:**

Fibrosis-5 (FIB-5) index is a marker of liver fibrosis and has been shown to have a good prognostic value for patients with acute heart failure (AHF), and C-reactive protein (CRP) has inflammatory properties and predicts adverse prognosis in patients with HF. However, the long-term prognostic value of FIB-5 index combined with CRP in patients with acute decompensated HF (ADHF) is yet unclear.

**Methods:**

This retrospective study included 1153 patients with ADHF hospitalized from January 2018 to May 2022.The FIB-5 index was calculated as (albumin [g/L]×0.3 + PLT count [10^9^/L]×0.05)−(ALP [U/L]×0.014 + AST to ALT ratio×6 + 14). Patients were stratified into the following four groups according to the median value of FIB-5 index (=-2.11) and CRP (= 4.5): Group 1 had a high FIB-5 index (FIB-5 index >-2.11) and a low CRP (CRP ≤ 4.5); Group 2 had both low FIB-5 index and low CRP; Group 3 had both high FIB-5 index and high CRP; Group 4 had a low FIB-5 index (FIB-5 index ≤-2.11) and a high CRP (CRP > 4.5). The endpoint was major adverse cardiac and cerebral events (MACCEs). Multivariate Cox analysis was used to evaluate the association of the combination with the development of MACCEs. Net reclassification improvement (NRI) and integrated discrimination improvement (IDI) analysis were used to compare the accuracy of the combination with a single prognostic factor for predicting the risk of MACCEs.

**Results:**

During the mean follow-up period of 584 ± 12 days, 488 (42.3%) patients had MACCEs. Kaplan–Meier analysis revealed that the incidence of MACCEs was different in the four groups (P < 0.001). After adjusting for the confounding factors, the hazard ratio (HR) for MACCEs in Group 4 (low FIB-5 index + high CRP) was the highest (Model 1, HR = 2.04, 95%CI 1.58–2.65, P < 0.001; Model 2, HR = 1.67, 95%CI 1.28–2.18, P < 0.001; Model 3, HR = 1.66, 95%CI: 1.27–2.17, P < 0.001). Additionally, the combination of FIB-5 index and CRP enabled more accurate prediction of MACCEs than FIB-5 index alone (NRI, 0.314,95%CI 0.199–0.429; P < 0.001; IDI, 0.023; 95% CI 0.015–0.032; P < 0.001).

**Conclusions:**

In patients with ADHF, the combination of the FIB-5 index and CRP may be useful in risk stratification in the future.

**Supplementary Information:**

The online version contains supplementary material available at 10.1186/s12872-023-03530-2.

## Introduction

Acute decompensated heart failure (ADHF) is the most common form of acute heart failure (AHF), accounting for about 70% of cases, and usually occurs in patients with a history of HF [1]. Patients with ADHF often require hospitalization for intravenous therapy due to worsening signs and symptoms of AHF caused by cardiac and vascular dysfunction and hemodynamic failure [2]. The high hospitalization and mortality rate in ADHF is a growing public health concern [3, 4]. Therefore, early identification of risk stratification markers in patients with ADHF plays a critical role in improving prognosis and therapeutic management.

Insufficient blood supply and circulatory congestion due to decreased cardiac output and increased circulatory resistance cause multiple organ dysfunction in the event of HF. A previous study showed that liver congestion and ischemia caused by acute and chronic heart failure exhibit abnormal liver-specific markers [5]. This interaction between the heart and liver is known as cardiohepatic syndrome [6, 7]. Abnormal liver function tests and markers of liver fibrosis are associated with poor prognoses in HF, such as reduced albumin(ALB) and increased bilirubin and transaminases [8, 9].

Presently, several liver function indicators have been published on the prediction model of HF risk [10, 11]. The fibrosis-4 (FIB-4) index is a simple marker evaluating liver fibrosis. It is calculated by age, transaminase level, and platelet (PLT) count [12] and can predict the adverse outcomes in HF with preserved ejection fraction (HFpEF) [13]. Recently, the fibrosis-5 (FIB-5) [14] index using albumin, alkaline phosphatase (ALP), aspartate aminotransferase(AST)/alanine aminotransferase (ALT) ratio, and PLT count has been shown to be superior to the FIB-4 index in predicting severe cirrhosis in patients with chronic hepatitis B and C [15, 16]. The FIB-5 index has better long-term prognostic value than the FIB-4 index as a valuable risk stratifier for the outcomes of cardiac death or rehospitalization in patients with AHF [17].

Inflammation is involved in the development of ADHF [2, 18]. C-reactive protein (CRP), produced by the liver, is an acute phase reactant and a non-specific marker of systemic inflammation, mainly regulated by interleukin-6 (IL-6) and tumor necrosis factor-alpha (TNF-α) [19, 20]. Some studies have shown that patients with high CRP levels have severe features of HF and are independently associated with mortality and morbidity [21]. CRP is a risk predictor for AHF and chronic HF [22–24].

However, the prognostic value of FIB-5 index combined with CRP for patients with ADHF has not been reported. Therefore, the present study aimed to investigate the predictive ability of FIB-5 index combined with CRP for adverse cardiovascular events in patients with ADHF and to verify whether FIB-5 index combined with CRP improves the predictive ability of adverse outcomes.

## Methods

### Study population

**Fig. 1:**
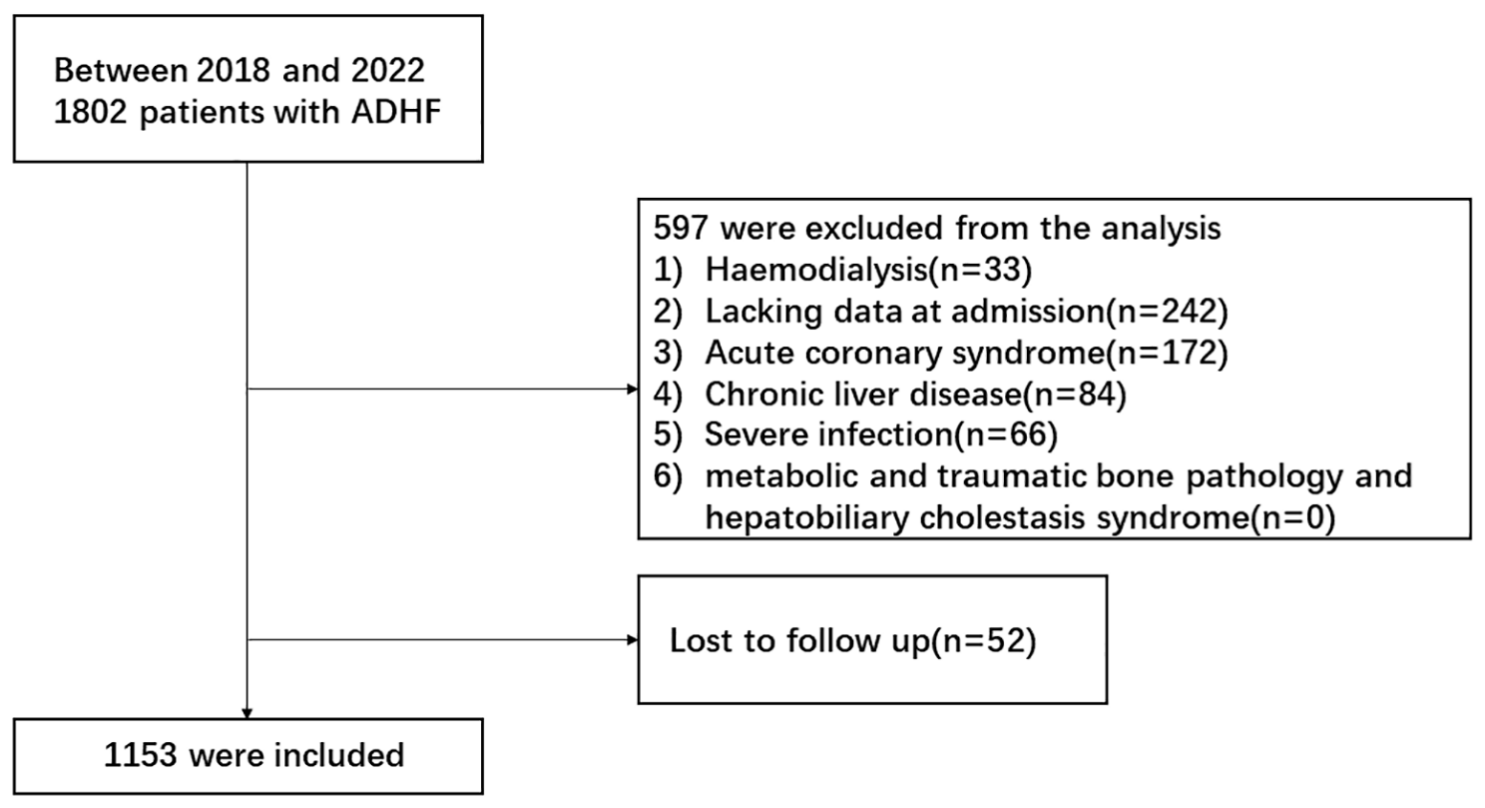
Flowchart of patient selection in this study. *ADHF* acute decompensated heart failure

This is a single-center, observational, and retrospective cohort study of 1802 consecutive ADHF patients admitted to Nanjing Drum Tower Hospital in Nanjing, China, from January 2018 to May 2022. The diagnosis of ADHF was based on the 2021 ESC guidelines for the diagnosis and treatment of acute and chronic heart failure [1], including the presence of symptoms or signs of heart failure (dyspnea) or signs (rales on chest X-ray, peripheral edema, etc.); NYHA functional class III or IV; evidence of systemic and/or diastolic dysfunction by echocardiography; BNP ≥ 100pg/mL or NT proBNP ≥ 300 pg/mL. The following data were extracted: (1) hemodialysis, (2) lacking data at admission, (3) acute coronary syndrome, (4) chronic liver diseases, (5) severe infection, (6) loss to follow-up, (7) metabolic and traumatic bone pathology (such as osteoporosis, Paget’s disease, fractures, etc.) and hepatobiliary cholestasis syndrome. Chronic liver diseases were defined as the presence of pre-existing liver disease and/or history of treatment based on the blood examination results and medical records reviewed by a hepatologist. Severe infections refer to infections caused by viruses, bacteria, and fungi, including bacterial pneumonia, urinary tract infections, biliary tract infections, sepsis, and other infections, as well as acute and chronic inflammatory syndromes. After excluding these patients, we studied 1153 patients with ADHF (mean age 69 ± 14; 469 men and 684 women) (Fig. 1). This retrospective study was conducted in line with the Declaration of Helsinki, with the approval from the ethics committee of Nanjing Drum Tower Hospital. Due to the retrospective nature of the study, the need for informed consent was waived by Nanjing Drum Tower Hospital ethical committee.

### Measurement and definitions of variables

The patient history, clinical examination, routine laboratory work-up, echocardiographic data, and medications during hospitalization were recorded. Philips IE33 ultrasound machine was used for echocardiography examination by an experienced echocardiographer. The echocardiographic parameters measured were interventricular septum thickness (IVSTD) (0.8-1.1 cm), left ventricular posterior wall thickness (LVPWT) (0.8-1.1 cm), left ventricular diastolic diameter (LVDd) (3.5-5.5 cm), aortic diameter (AoD) (2.5-3.7 cm), left atrial diameter (LAD) (2.7-3.7 cm), and left ventricular ejection fraction (LVEF) (50–70%). We measured left ventricular ejection fraction (LVEF) using the modified Simpson technique.

Diabetes mellitus (DM) was defined as fasting plasma glucose (FPG) ≥ 7mmol/L or hemoglobin A1c (HbA1c) ≥ 6.5%, or a history of diabetes. Hypertension was defined as systolic blood pressure (SBP) ≥ 140 mmHg and/or diastolic blood pressure (DBP) ≥ 90 mmHg, or the use of antihypertensive drugs. Body mass index (BMI) was calculated as the patient’s weight in kilograms divided by their height in meters squared. According to WHO criteria, defined as: underweight (< 18.5 kg/m2), normal weight (18.5–24.9 kg/m2), overweight (25.0–29.9 kg/m2), obesity (≥ 30 kg/m2). Dyslipidemia was defined as TC ≥ 200 mg/dL, TG ≥ 150 mg/dL, LDL-C ≥ 100 mg/dL, HDL < 400 mg/dL for men and < 500 mg/dL for women or the use of a lipid-lowering drug (even in the presence of normal lipid levels). Any of the above conditions are considered dyslipidemia.

The use of statins, antiplatelet agents, aldosterone antagonists, beta-blockers, diuretics, angiotensin-converting enzyme inhibitor (ACEI)/angiotensin receptor inhibitors (ARB)/angiotensin receptor neprilysin inhibitor (ARNI), digoxin, sodium-glucose cotransporter-2 inhibitor (SGLT2i), and insulin was recorded.

The participant’s blood samples were collected in the early morning after overnight fasting, including CRP(0-8 mg/L), B-type natriuretic peptide (BNP) (5-100 pg/mL), hematocrit(40–50 %), ALB(40-55 g/L), uric acid (UA)(90–420 µmol/L), creatinine(44–106 µmol/L), total bilirubin(5.1–28.0 µmol/L), serum sodium(135-145 mmol/L), serum potassium(3.5-5.5 mmol/L), white blood cell (WBC)(3.5–9.5 10^9^/L), prothrombin time (10-15 s), PLT(125–350 10^9^/L), ALT(5-40 U/L), AST(8-40 U/L), lactate dehydrogenase(109-245 U/L), ALP(47-185 U/L), total cholesterol(112.11-221.14 mg/dL), triglycerides(49.62-150.62 mg/dL), high-density lipoprotein cholesterol (HDL-C)(36.34 -77.32 mg/dL), low-density lipoprotein cholesterol (LDL-C) (73.07-119.85 mg/dL), glycated hemoglobin (HbA1c)(4.2-6.0 %), fasting plasma glucose (FPG)(3.9-6.1 mmol/L), hemoglobin(130-175 g/L), and blood urea nitrogen (BUN)(3.2-7.1 mmol/L).

### Fibrosis-5 index calculation

FIB-5 index = (albumin [g/L] × 0.3 + PLT count [10^9^/L] × 0.05) - (ALP [U/L] × 0.014 + AST to ALT ratio × 6 + 14) [14].

### Clinical outcomes and follow up

The endpoint of this study was defined as MACCEs, which is the composite outcome of all-cause death, non-fatal stroke, non-fatal myocardial infarction and revascularization, malignant arrhythmia, and requiring hospitalization due to worsening heart failure. After discharge, all patients were followed up by telephone or outpatient clinic visits by hospital-trained physicians every 6 months. The mean follow-up time was 584 ± 12 days and the total duration is 5 years.

### Statistical analysis

For quantitative variables, normally distributed data are expressed as mean ± standard deviation (SD), and non-normally distributed data are presented as median [interquartile range (IQR)]. Categorical variables are expressed as numbers and percentages. Two-group comparisons were analyzed by the Student’s t-test or the Mann–Whitney u-test for continuous variables. Continuous variables were compared by analysis of variance (ANOVA) or the Kruskal–Wallis test among the four groups. χ2 test was used to compare the differences in categorical variables.

The event-free survival rates were calculated using the Kaplan–Meier method, and the differences in survival rates were compared between groups with the log-rank test. The prognostic value of the baseline characteristics was assessed with Cox proportional hazards regression analysis. The baseline variables with P < 0.1 or clinical significance were selected and included in the Cox proportional hazards models. Finally, three multivariable regression models were established: Model 1, adjustment for age, gender, systolic blood pressure, diastolic blood pressure, heart rate, and BMI; Model 2, adjustment for variables included in Model 1 plus BNP, hematocrit, uric acid, white blood cell, creatinine, serum sodium, serum potassium, LDL-C, left atrial diameter, and LVEF; Model 3, adjustment for variables included in Model 2 plus history of hypertension, valvular heart disease, atrial fibrillation, use of aldosterone antagonist, beta-blocker, diuretic, statin, digoxin, angiotensin-converting enzyme inhibitor (ACEI)/angiotensin receptor inhibitors (ARB)/angiotensin receptor neprilysin inhibitor (ARNI), and sodium-glucose contransporter-2 inhibitor (SGLT2i). Hazard ratios (HRs) were calculated, and the results were reported as HRs and 95% confidence intervals (CIs). Also, to measure the improvement in predictive accuracy attained by adding a new variable to the variable of the FIB-5 index or CRP, the continuous net reclassification improvement (NRI) and integrated discrimination improvement (IDI) were analyzed to evaluate the prognostic value of FIB-5 index combined with CRP compared to independent models in patients with ADHF. We also conducted subgroup analyses on variables, including age, sex, BMI, LVEF, history of hypertension, and history of diabetes mellitus. Interactions between subgroups were tested in the multivariate Cox proportional risk regression model.

All data were analyzed with R version 4.2.1 and SPSS for Windows version 26, and P < 0.05 was considered statistically significant.

## Results

### Baseline characteristics of the study population

The clinical characteristics of the 1153 patients with ADHF are summarized in Table 1. The mean follow-up time was 584 ± 12 days. The mean age of the participants was 69 ± 14 years, 59.3% were females, and 488 patients (42.3%) fulfilled the primary composite outcome of MACCEs. In the population with a positive outcome, FIB-5 index was lower, while heart rate, CRP, BNP, UA, creatinine, prothrombin time, AST, lactate dehydrogenase, and BUN were higher. Patients with MACCEs were significantly older and had higher blood sugar levels, such as HbA1C and FPG. Compared with the non-MACCEs group, diabetics were more prevalent in the MACCEs group (43% Vs. 35%, p = 0.014). We found that the proportions of patients with hypoHDLemia were significantly higher in the MACCEs group. Patients with MACCEs were less likely to use of antiplatelet agent, ACEI/ARB/ARNI, and SGLT2i than those with non-MACCEs.

**Table 1:** Baseline clinical characteristics

	Overall (n = 1153)	Non-MACCEs (n = 665)	MACCEs (n = 488)	P-value
Female, n (%)	684 (59.32)	394 (59.25)	290 (59.43)	0.952
Age (years)	68.7 ± 14.2	67.4 ± 14.5	70.5 ± 14.3	< 0.001
SBP (mmHg)	130.0 ± 22.9	130.0 ± 22.3	130.0 ± 23.9	0.979
DBP (mmHg)	76.2 ± 15.4	77.3 ± 15.2	74 0.7 ± 15.5	0.010
Heart rate (bpm)	83.4 ± 22.1	82.1 ± 21.6	85.1 ± 22.7	0.021
BMI, n (%)				0.227
< 18.5 (kg/m^2^)	79(6.85)	42(6.32)	37(7.58)	0.400
18.5–24.9 (kg/m^2^)	566(49.09)	315(47.37)	251(51.43)	0.148
25-29.9 (kg/m^2^)	397(34.43)	245(36.84)	152(31.15)	0.065
≥ 30 (kg/m^2^)	111(9.63)	63(9.47)	48(9.84)	0.928
Valvular heart disease, n (%)	240 (20.82)	136 (20.45)	104 (21.31)	0.778
Atrial fibrillation, n (%)	502 (43.54)	279 (41.95)	223 (45.70)	0.228
Dyslipidemia, n (%)	947(82.13)	540(81.20)	407(83.40)	0.671
Hypercholesterolemia, n (%)	118(10.23)	76(11.43)	42(8.61)	0.118
Hypertriglyceridemia, n (%)	209(18.13)	128(19.25)	81(16.60)	0.249
hypoHDLemia, n (%)	830(71.99)	461(69.32)	369(75.61)	0.019
Hypertension, n (%)	690 (59.84)	387 (58.20)	303 (62.09)	0.203
Diabetes mellitus, n (%)	450 (39.03)	239 (35.94)	211 (43.24)	0.014
BNP (pg/mL)	492 (212–1093)	430 (194–891)	647 (268–1323)	< 0.001
CRP (mg/L)	4.5 (2.9–11.0)	4.0 (2.7–7.9)	5.7 (3.3–19.5)	< 0.001
FIB-5 index	-2.11 (-6.04-0.98)	-1.68 (-5.25-1.31)	-2.96 (-7.28-0.60)	< 0.001
Hematocrit (%)	38.1 ± 7.1	39.3 ± 8.1	36.4 ± 6.9	< 0.001
ALB (g/L)	37.81 ± 3.9	38.5 ± 3.6	36.8 ± 4.2	< 0.001
UA (µmol/L)	447.3 ± 150.6	436.1 ± 141.8	462.5 ± 160.6	0.005
Creatinine (µmol/L)	83.0 (66.0-110.0)	80.0 (64.0-102.0)	88.1 (70.0-126.5)	< 0.001
Total bilirubin (µmol/L)	13.0 (9.0-19.1)	13.1 (9.4–18.8)	13.0 (8.8–19.4)	0.636
Sodium (mmol/L)	138.5 ± 4.3	138.8 ± 3.9	138.1 ± 4.5	0.003
Potassium (mmol/L)	3.9 ± 0.6	3.9 ± 0.5	4.0 ± 0.6	0.848
WBC (10^9^/L)	6.1 (5.0-7.7)	6.1(5.0-7.6)	6.3(5.0-8.2)	0.001
Prothrombin time (s)	12.2 (11.3–13.5)	12.0 (11.2–13.1)	12.4 (11.6–14.0)	< 0.001
PLT (10^9^/L)	172.0 (135.0-216.0)	173.0 (140.0-214.0)	170.0 (125.8–221.0)	0.277
ALT (U/L)	19.4 (13.0-33.2)	19.7 (13.1–33.3)	19.0 (12.5–32.8)	0.495
AST (U/L)	23.3 (17.5–33.2)	22.8 (17.0-32.7)	24.0 (18.4–35.1)	0.034
Lactate dehydrogenase (U/L)	241.0 (198.0-328.0)	231.0 (193.0-297.3)	259.0 (210.0-391.0)	< 0.001
ALP (U/L)	71.5 (57.9–89.9)	71.1 (57.9–89.4)	72.3 (58.0-90.6)	0.753
Total cholesterol (mg/dL)	139.98 (115.24-171.02)	142.88 (116.00-174.30)	137.28 (111.37-166.76)	0.006
Triglyceride (mg/dL)	88.57 (68.20-127.55)	90.35 (69.31-131.98)	88.57(66.43 -119.57)	0.026
HDL-C (mg/dL)	38.67(30.94–46.40)	38.67 (32.48–47.56)	36.93 (28.62–44.47)	< 0.001
LDL-C (mg/dL)	77.34 (57.23-104.41)	77.73 (59.16-107.12)	77.34 (55.30-100.15)	0.047
HbA1C (%)	6.5 ± 1.4	6.4 ± 1.4	6.6 ± 1.5	0.030
FPG (mmol/L)	5.8 ± 2.2	5.6 ± 1.8	6.0 ± 2.5	0.032
Hemoglobin (g/L)	126.1 ± 24.5	130.3 ± 23.6	120.4 ± 24.6	< 0.001
BUN (mmol/L)	7.6 (5.8–10.0)	7.0 (5.6–9.1)	8.0 (6.0–12.0)	< 0.001
IVSTD (cm)	0.9 (0.8-1.0)	0.9 (0.8-1.0)	0.9 (0.8-1.0)	0.347
LVPWT (cm)	0.9 (0.8-1.0)	0.9 (0.8-1.0)	0.9 (0.8-1.0)	0.281
LVDd (cm)	5.8 (5.1–6.6)	5.8 (5.2–6.6)	5.8 (5.1–6.5)	0.252
AoD (cm)	3.3 (3.0-3.5)	3.3 (3.0-3.4)	3.2 (3.0-3.5)	0.690
LAD (cm)	4.8 (4.4–5.3)	4.8 (4.4–5.3)	4.8 (4.4–5.3)	0.361
LVEF (%)	40 (31–53)	40 (30–52)	40 (31–54)	0.345
Medications at admission, n (%)				
Statins	643 (55.77)	382 (57.44)	261 (53.48)	0.201
Antiplatelet agent	546 (47.35)	333 (50.08)	213 (43.65)	0.036
Aldosterone antagonist	514 (44.58)	303 (45.56)	211 (43.24)	0.468
Beta-blocker	884 (76.67)	511 (76.84)	373 (76.43)	0.927
Diuretics	1008 (87.42)	577 (86.77)	431 (88.32)	0.487
ACEI/ARB/ARNI	481 (41.75)	310 (46.69)	171 (35.04)	< 0.001
Digoxin	137 (11.89)	67 (10.09)	70 (14.34)	0.035
SGLT2i	91 (7.90)	68 (10.24)	23 (4.71)	0.001
Insulin	144 (12.50)	84 (12.65)	60 (12.30)	0.928

*SBP* systolic blood pressure; *DBP* diastolic blood pressure; *BMI* body mass index; *FIB-5* fibrosis-5; *CRP* C-reactive protein; *ALB* albumin; *ALP* alkaline phosphatase; *ALT* alanine aminotransferase; *AST* aspartate aminotransferase; *PLT* platelet; *BNP* B-type natriuretic peptide; *UA* uric acid; *WBC* white blood cell; *IVSTD* interventricular septum thickness; *LVPWT* left ventricular posterior wall thickness; *LVDd* left ventricular diastolic diameter; *AoD* aortic diameter; *LAD* left atrial diameter; *LVEF* left ventricular ejection fraction; *HDL-C* high-density lipoprotein cholesterol; *LDL-C* low-density lipoprotein cholesterol; *HbA1c* glycated hemoglobin; *FPG* fasting plasma glucose; *BUN* blood urea nitrogen; *ACEI* angiotensin-converting enzyme inhibitor; *ARB* angiotensin receptor inhibitors; *ARNI* angiotensin receptor neprilysin inhibitor; *SGLT2i* sodium-glucose cotransporter-2 inhibitor; *MACCEs* major adverse cardiac and cerebral events

### Baseline characteristics of groups of FIB5-CRP

The patients were divided into the following four groups according to the median value of FIB-5 index (=-2.11) and CRP (= 4.5): Group 1 had a high FIB-5 and a low CRP (FIB-5 index >-2.11, CRP ≤ 4.5); Group 2 had a low FIB-5 index and a low CRP (FIB-5 index ≤-2.11, CRP ≤ 4.5); Group 3 had a high FIB-5 index and a high CRP (FIB-5 index >-2.11, CRP > 4.5); Group 4 had a low FIB-5 index and a high CRP (FIB-5 index ≤-2.11, CRP > 4.5).

BNP, UA, creatinine, total bilirubin, prothrombin time, AST, ALP, Lactate dehydrogenase, BUN and LVEF were higher in Group 4 compared to Group 1, Group 2, and Group 3. However, no difference was detected in SBP, BMI, IVSTD, LVPWT, the occurrence of valvular heart disease and hypertension, the use of aldosterone antagonist, beta-blocker, diuretics, ACEI/ARB/ARNI, Digoxin, SGLT2i, and insulin in the four groups. The other characteristics are listed in Table 2.

**Table 2:** Clinical characteristics of different groups of FIB5-CRP

	Group 1 (n = 309)	Group 2 (n = 275)	Group 3 (n = 268)	Group 4 (n = 301)	P-value
Female, n (%)	186 (60.19)	156 (56.73)	156 (58.21)	186 (61.79)	0.623
Age (years)	64.9 ± 14.1	72.5 ± 12.3	66.1 ± 16.7	71.3 ± 13.4	< 0.001
SBP (mmHg)	127.8 ± 20.0	132.5 ± 23.2	130.1 ± 23.3	130.1 ± 25.3	0.145
DBP (mmHg)	77.2 ± 14.4	75.2 ± 16.4	77.7 ± 15.4	74.7 ± 15.5	0.044
Heart rate (bpm)	81.5 ± 20.3	77.7 ± 20.4	87.6 ± 23.4	87.1 ± 23.1	< 0.001
BMI, n (%)					0.647
< 18.5 (kg/m^2^)	18(5.83)	19(6.91)	15(5.60)	27(8.97)	0.349
18.5–24.9 (kg/m^2^)	145(46.93)	134(48.73)	134(50.00)	153(50.83)	0.861
25-29.9 (kg/m^2^)	109(35.28)	97(35.27)	94(35.07)	97(32.23)	0.884
≥ 30 (kg/m^2^)	37(11.97)	25(9.09)	25(9.33)	24(7.97)	0.470
Valvular heart disease, n (%)	57 (18.45)	69 (25.09)	52 (19.40)	62 (20.60)	0.218
Atrial fibrillation, n (%)	104 (33.66)	145 (52.73)	108 (40.30)	145 (48.17)	< 0.001
Dyslipidemia, n (%)	257(83.17)	197(71.64)	233(86.94)	259(86.05)	< 0.001
Hypercholesterolemia, n (%)	38(12.30)	18(6.55)	37(13.81)	25(8.31)	0.015
Hypertriglyceridemia, n (%)	89(28.80)	32(11.64)	48(17.91)	40(13.29)	< 0.001
hypoHDLemia, n (%)	215(69.58)	174(63.27)	209(77.99)	232(77.08)	< 0.001
Hypertension, n (%)	178 (57.61)	162 (58.91)	165 (61.57)	185 (61.46)	0.705
Diabetes mellitus, n (%)	110 (35.60)	99 (36.00)	128 (47.76)	113 (37.54)	0.010
BNP (pg/mL)	393 (160–779)	421 (206–918)	541 (239–1198)	781 (332–1343)	< 0.001
Hematocrit (%)	40.1 ± 5.9	37.6 ± 6.5	38.5 ± 6.7	36.0 ± 10.4	< 0.001
ALB (g/L)	39.7 ± 3.1	37.9 ± 3.8	37.9 ± 3.7	35.6 ± 4.1	< 0.001
UA ((µmol/L)	432.4 ± 142.6	432.8 ± 139.9	461.3 ± 165.2	464.3 ± 153.5	0.012
Creatinine (µmol/L)	78 (63–97)	84 (68–107)	82 (67–110)	89 (68–132)	0.001
Total bilirubin (µmol/L)	12.0 (8.8–16.7)	13.4 (9.5–21.1)	12.7 (9.1–18.3)	15.0 (9.1–22.0)	0.001
Sodium (mmol/L)	139.1 ± 3.8	138.8 ± 4.1	138.2 ± 4.4	137.7 ± 4.7	< 0.001
Potassium (mmol/L)	4.0 ± 0.5	3.9 ± 0.6	3.9 ± 0.6	3.8 ± 0.6	0.036
WBC (10^9^/L)	6.4 (5.5–7.7)	5.3 (4.3–6.3)	7.0 (5.6-9.0)	6.1 (5.0–8.0)	< 0.001
Prothrombin time (s)	11.7 (10.9–12.7)	12.3 (11.5–13.9)	12.0 (11.3–13.1)	12.8 (11.8–14.3)	< 0.001
PLT (10^9^/L)	196 (168–231)	138 (108–164)	213 (176–254)	144 (111–177)	< 0.001
ALT (U/L)	24.6 (16.9–38.0)	14.2 (10.1–20.8)	26.8 (16.8–44.0)	16.3 (10.8–26.5)	< 0.001
AST (U/L)	22.1 (17.3–30.0)	22.4 (17.1–31.5)	23.2 (17.1–35.2)	25.1 (19.0–40.0)	< 0.001
Lactate dehydrogenase (U/L)	218 (183–261)	243 (195–335)	236 (198–298)	302 (231–482)	< 0.001
ALP(U/L)	66.2 (54.3–80.1)	69.4 (55.6–86.7)	73.7(60.3–91.6)	80.0 (64.4-103.3)	< 0.001
Total cholesterol (mg/dL)	147.91 (122.10-183.39)	136.89 (115.24-156.61)	145.01 (116.01-175.56)	129.93 (109.34-165.41)	< 0.001
Triglyceride (mg/dL)	104.96(79.72-159.88)	85.03 (58.46-107.17)	92.12 (73.30-131.31)	87.25 (62.67-112.49)	< 0.001
HDL-C (mg/dL)	38.67(32.10-47.56)	39.83(34.03–51.43)	35.58 (28.62–42.54)	36.74 (29.00-44.08)	< 0.001
LDL-C (mg/dL)	84.88 (63.13-108.66)	74.63 (54.52–93.19)	80.82(61.10-116.01)	71.93 (51.82-100.35)	< 0.001
HbA1C (%)	6.4 ± 1.3	6.3 ± 1.2	6.8 ± 1.6	6.5 ± 1.5	0.001
FPG (mmol/L)	5.6 ± 1.9	5.4 ± 1.7	6.3 ± 2.6	5.9 ± 2.3	< 0.001
Hemoglobin (g/L)	133.4 ± 20.8	125.2 ± 23.7	127.9 ± 23.7	117.6 ± 27.0	< 0.001
BUN (mmol/L)	7.0 (5.8–8.8)	7.7 (5.8–10.0)	7.2 (5.5–10.2)	8.1 (6.0–13.0)	< 0.001
IVSTD (cm)	0.9 (0.8-1.0)	0.9 (0.8-1.0)	0.9 (0.8-1.0)	0.9 (0.8-1.0)	0.325
LVPWT (cm)	0.9 (0.8-1.0)	0.9 (0.8-1.0)	0.9 (0.8-1.0)	0.9 (0.8-1.0)	0.474
LVDd (cm)	6.1 (5.4–6.8)	5.8 (5.0-6.5)	6.0 (5.3–6.7)	5.6 (5.0-6.2)	< 0.001
AoD (cm)	3.2 (3.0-3.4)	3.3 (3.1–3.5)	3.2 (3.0-3.4)	3.3 (3.0-3.5)	0.043
LAD (cm)	4.8 (4.4–5.2)	5.0 (4.5–5.5)	4.8 (4.4–5.2)	4.8 (4.3–5.2)	< 0.001
LVEF (%)	38 (29–52)	43 (33–54)	38 (29–52)	44 (35–54)	< 0.001
Medications at admission, n (%)					
Statins	198 (64.08)	145 (52.73)	141 (52.61)	159 (52.82)	0.008
Antiplatelet agent	167 (54.05)	122 (44.36)	127 (47.39)	130 (43.19)	0.035
Aldosterone antagonist	133 (43.04)	111 (40.36)	124 (46.27)	146 (48.50)	0.216
Beta-blocker	237 (76.70)	198 (72.00)	217 (80.97)	232 (77.08)	0.105
Diuretics	265 (85.76)	245 (89.09)	230 (85.82)	268 (89.04)	0.422
ACEI/ARB/ARNI	143 (46.28)	111 (40.36)	116 (43.28)	111 (37.00)	0.118
Digoxin	41 (13.27)	29 (10.55)	33 (12.31)	34 (11.33)	0.760
SGLT2i	23 (7.44)	19 (6.91)	24 (8.96)	25 (8.33)	0.814
Insulin	34 (11.00)	35 (12.73)	39 (14.55)	36 (12.00)	0.627

Group1: high FIB5 + low CRP; Group2: low FIB5 + low CRP; Group3: high FIB5 + high CRP; Group4: low FIB5 + high CRP

*SBP* systolic blood pressure; *DBP* diastolic blood pressure; *BMI* body mass index; *FIB-5* fibrosis-5; *CRP* C-reactive protein; *ALB* albumin; *ALP* alkaline phosphatase; *ALT* alanine aminotransferase; *AST* aspartate aminotransferase; *PLT* platelet; *BNP* B-type natriuretic peptide; *UA* uric acid; *WBC* white blood cell; *IVSTD* interventricular septum thickness; *LVPWT* left ventricular posterior wall thickness; *LVDd* left ventricular diastolic diameter; *AoD* aortic diameter; *LAD* left atrial diameter; *LVEF* left ventricular ejection fraction; *HDL-C* high-density lipoprotein cholesterol; *LDL-C* low-density lipoprotein cholesterol; *HbA1c* glycated hemoglobin; *FPG* fasting plasma glucose; *BUN* blood urea nitrogen; *ACEI* angiotensin-converting enzyme inhibitor; *ARB* angiotensin receptor inhibitors; *ARNI* angiotensin receptor neprilysin inhibitor; *SGLT2i* sodium-glucose cotransporter-2 inhibitor

### Predictive ability of the FIB5-CRP groups for MACCEs

**Fig. 2:**
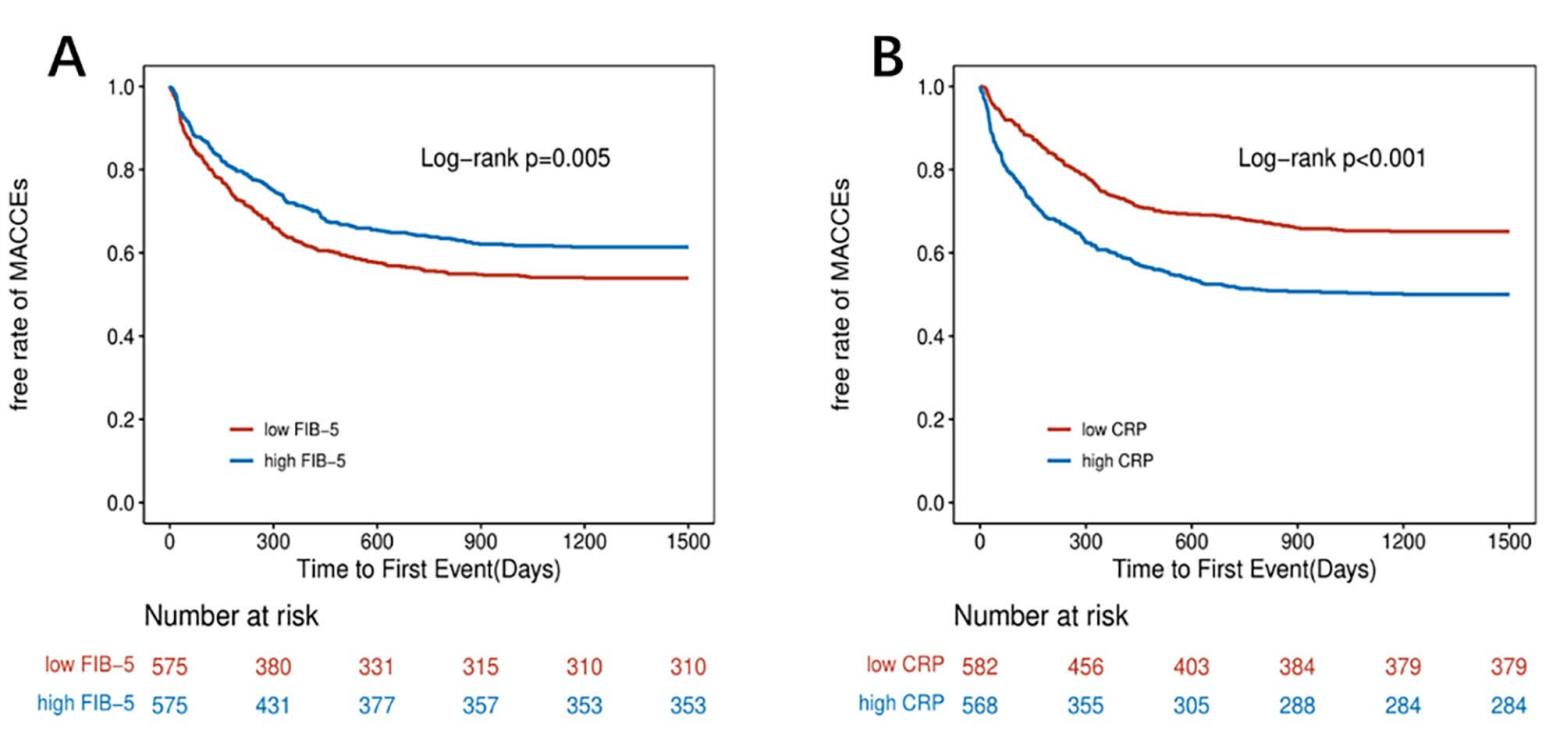
Kaplan–Meier analysis of MACCEs according to FIB-5 index (**A**) and CRP (**B**). Patients with ADHF were stratified by the median value of the model for FIB-5 index and CRP (overall cohort). *FIB-5* fibrosis-5; *ADHF* acute decompensated heart failure; *CRP* C-reactive protein; *MACCEs* major adverse cardiac and cerebral events

The event-free survival for MACCEs according to the FIB-5 index and CRP median value using Kaplan–Meier curves is presented in Fig. 2. Compared with patients with high FIB-5 index, the cumulative incidence of MACCEs in patients with low FIB-5 index was significantly higher (Fig. 2A). Conversely, in Fig. 2B, Kaplan–Meier analysis showed that the high CRP group have a significantly greater risk of MACCEs, compared with the low CRP group (P < 0.05, log-rank test).

The interaction between the FIB-5 index and CRP was not statistically significant (P = 0.98), indicating that the correlation between the FIB-5 index and MACCEs risk does not vary with CRP levels.

**Fig. 3:**
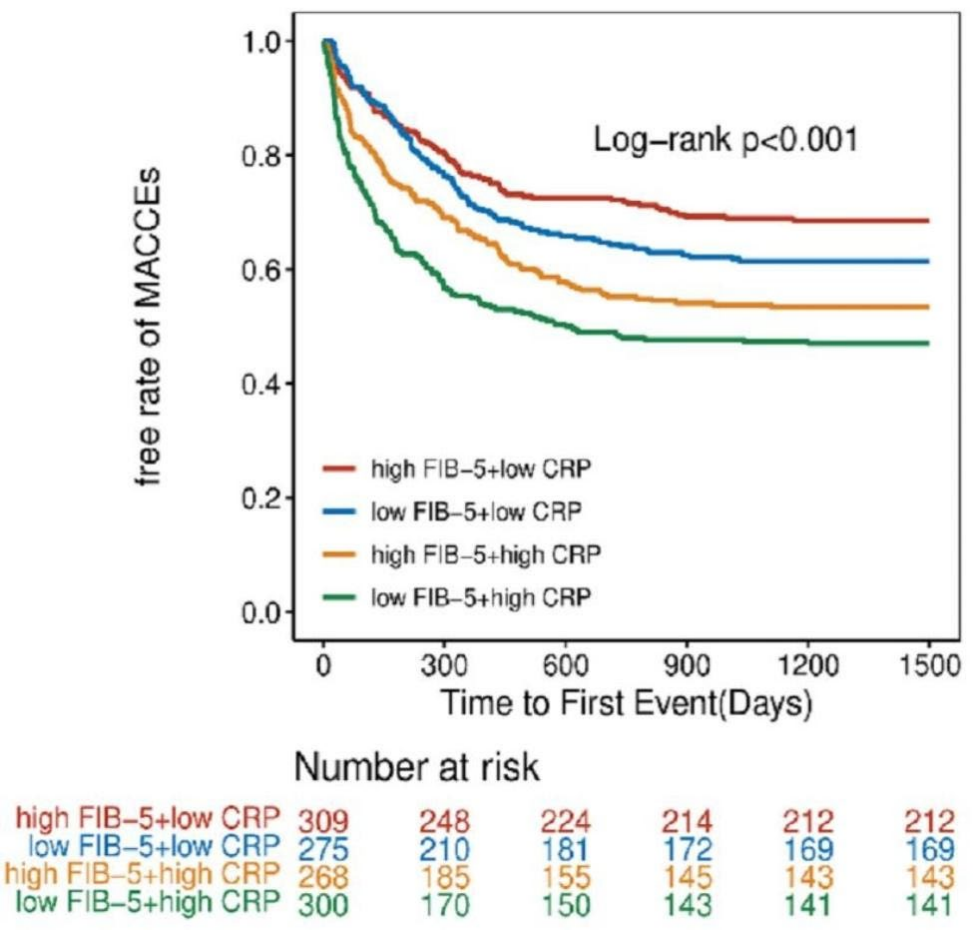
Kaplan–Meier analysis of MACCEs in the FIB5-CRP groups. *FIB-5* fibrosis-5; *CRP* C-reactive protein; *MACCEs* major adverse cardiac and cerebral events

On the other hand, a significant difference was observed in the risk of MACCEs in the FIB-5 index combined with CRP groups (Fig. 3). The Kaplan–Meier analysis revealed that patients with low FIB-5 and high CRP had the lowest event-free rate on MACCEs events (P < 0.001, log-rank test).

**Fig. 4:**
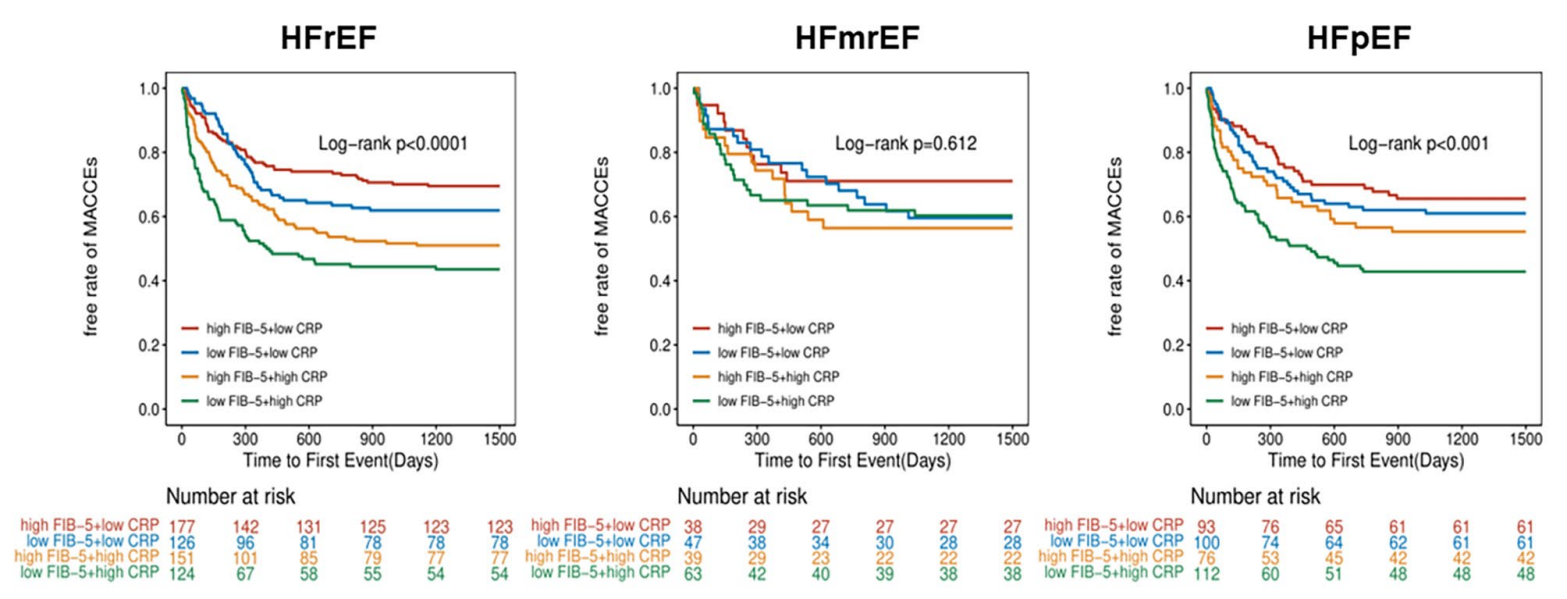
Kaplan–Meier analysis of MACCEs with HFrEF (LVEF < 40%), HFmrEF(LVEF 40–49%), and HFpEF(LVEF ≥ 50%). *HFpEF* Heart Failure with preserved ejection fraction; *HFrEF* Heart Failure with reduced ejection fraction; *HFmrEF* Heart Failure with mid-range ejection fraction; *MACCEs* major adverse cardiac and cerebral events

When patients were divided into groups with HF with reduced ejection fraction (HFrEF) (LVEF < 40%), HF with mid-range ejection fraction (HFmrEF) (LVEF 40–49%), and HFpEF (LVEF ≥ 50%), the endpoint results were consistent with those of the groups with HFrEF and HFpEF (P < 0.001, log-rank test). However, in the HFmrEF group, patients with a low FIB-5 index and high CRP did not exhibit a significantly increased risk of MACCEs (P = 0.612, log-rank test) (Fig. 4).

In the current data, the events of MACCEs were recorded in 97 (8.4%) cases in Group 1, 106 (9.2%) in Group 2, 125 (10.8%) in Group 3, and 160 (13.9%) in Group 4 (Table 3). In multivariate Cox proportional hazards analysis, FIB-5 index as a continuous variable also can independently predict the occurrence of MACCEs (Additional File Table 3). Group 4 (low FIB-5 and high CRP) is associated with a high incidence of MACCEs (Model 1, HR = 2.04, 95% CI: 1.58–2.65, P < 0.001; Model 2, HR = 1.67, 95% CI: 1.28–2.18, P < 0.001; Model 3, HR = 1.66, 95% CI: 1.27–2.17, P < 0.001), while no significant differences were observed in Group 2 (low FIB-5 and low CRP) (Table 3).

**Table 3:** HR (95% CI) of MACCEs according to FIB5-CRP group in the three models

**Groups**	**Events, n (%)**	Model 1		Model 2		Model 3	
		HR (95% CI)	P-value	HR (95% CI)	P-value	HR (95% CI)	P-value
high FIB-5 and low CRP	97 (8.4)	Ref.		Ref.		Ref.	
low FIB-5 and low CRP	106 (9.2)	1.21 (0.91–1.60)	0.183	1.13 (0.85–1.50)	0.412	1.12 (0.84–1.49)	0.438
high FIB-5 and high CRP	125 (10.8)	1.68 (1.28–2.19)	< 0.001	1.40 (1.06–1.83)	0.017	1.39 (1.06–1.83)	0.017
low FIB-5 and high CRP	160 (13.9)	2.04 (1.58–2.65)	< 0.001	1.67 (1.28–2.18)	< 0.001	1.66 (1.27–2.17)	< 0.001

*HR* hazard ratio; *CI* confidence intervals; *FIB-5* fibrosis-5; *CRP* C-reactive protein;

Model 1: adjusted for age, gender, SBP, DBP, heart rate, and BMI.

Model 2: adjusted for Model 1 + BNP, hematocrit, UA, WBC, Creatinine, Sodium, Potassium, LDL-C, LAD, and LVEF.

Model 3: adjusted for Model 2 + history of hypertension, valvular heart disease, atrial fibrillation, and use of aldosterone antagonist, Beta-Blockers, diuretics, statins, digoxin, ACEI/ARB/ARNI, and SGLT2i

### Predictive efficacy of FIB5-CRP model on MACCEs in patients with ADHF

Compared to the single CRP model, the NRI and IDI of CRP combined with the FIB-5 index were significantly increased (NRI = 0.158, 95% CI: 0.041–0.027, P = 0.008; IDI = 0.006, 95% CI: 0.001–0.010, P = 0.009), and the NRI and IDI of FIB-5 index combined with CRP were also significantly increased when compared to the single FIB-5 index model (NRI = 0.314, 95% CI: 0.199–0.429, P < 0.001; IDI = 0.023,95% CI: 0.015–0.032, P < 0.001) (Table 4).

**Table 4:** The predicted performance of the FIB5-CRP model

	NRI (95% CI)	P-value	IDI (95% CI)	P-value
FIB5 + CRP vs. CRP	0.158 (0.041–0.274)	0.008	0.006 (0.001–0.010)	0.009
FIB5 + CRP vs. FIB5	0.314 (0.199–0.429)	< 0.001	0.023 (0.015–0.032)	< 0.001

*FIB-5* fibrosis-5; *CRP* C-reactive protein; *CI* confidence interval; *NRI* net reclassification improvement; *IDI* integrated discrimination improvement

### Subgroup analysis

**Fig. 5:**
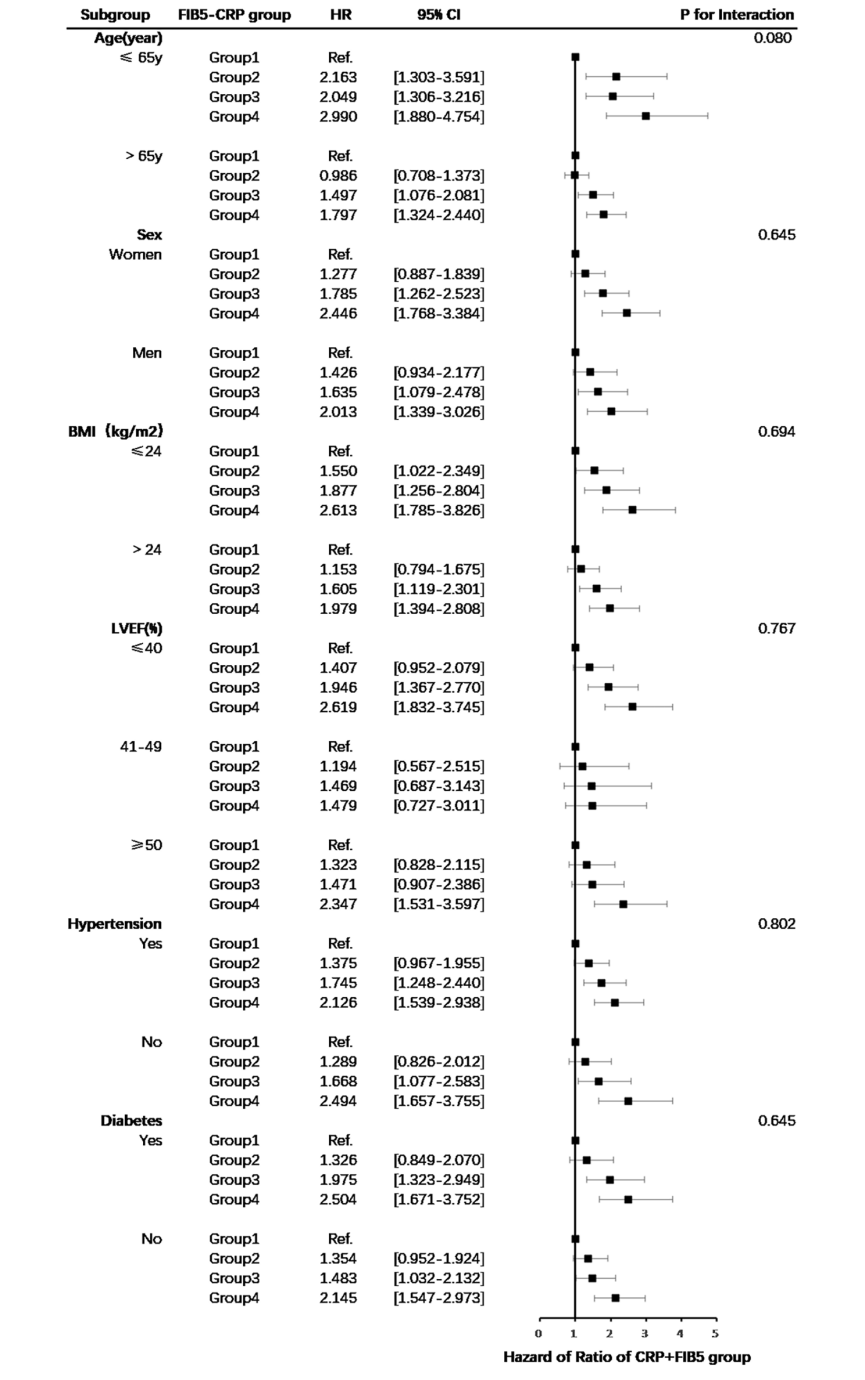
Forest plot of MACCEs according to different subgroups. Group1: high FIB5 + low CRP; Group2: low FIB5 + low CRP; Group3: high FIB5 + high CRP; Group4: low FIB5 + high CRP; *MACCEs* major adverse cardiac and cerebral events

The subgroup analysis showed that the associations of the FIB5-CRP groups with the risk of MACCEs were consistent across the subgroups with respect to age, sex, BMI, LVEF, history of hypertension, and diabetes mellitus (Fig. 5). Group 4 was associated with a higher incidence of MACCEs compared to the other groups. Moreover, no significant interactions were observed between the subgroup factors and the FIB5-CRP groups for MACCEs (P-values for interaction > 0.05).

## Discussion

In this study, patients with ADHF in the low FIB5 and high CRP group were at a significantly increased risk of adverse outcomes, while the high FIB5 and low CRP group had the lowest risk of MACCEs. The event-free rate was lower in both the high FIB5 and CRP group than in the low FIB5 and CRP group. The association was consistent in both patients with HFpEF and HFrEF except in patients with HFmrEF. In addition, FIB-5 index combined with CRP could also be used for risk stratification when analyzing subgroups such as age, sex, BMI, LVEF, history of hypertension, and diabetes mellitus. We found that FIB-5 index combined with CRP added incremental prognostic information provided by FIB-5 index or CRP alone. The combination further enhanced the predictive power of poor prognosis in patients with ADHF. As far as we know, this is the first study to show that the combination of FIB-5 index and CRP may be useful in stratifying patients with ADHF at risk of developing MACCEs.

### Association between the FIB-5 index and cardiovascular disease

In ADHF, two models have been proposed for the pathophysiological mechanism between HF and hepatic insufficiency [10, 25–28]: (1) A significant increase in vena cava and central venous pressure can be transmitted to the central hepatic sinusoids of the hepatic lobules to compress the intrahepatic bile ducts and tubules, redirecting the bile flow and increasing the ALP and glutamyl transferase levels. The increase in vena cava and central venous pressure also stagnates blood flow to the liver, favoring the formation of thrombi in the hepatic sinusoids, hepatic veins, and portal veins, thereby reducing the platelets. (2) Decreased cardiac output reduces liver perfusion, allowing damage and necrosis to the central hepatocytes of the hepatic lobules, which in turn increases bilirubin and transaminases. The fact that liver cells in the central lobular area contain higher levels of AST than ALT, supports the result of an increase in transaminase levels led by AST in ADHF patients. The FIB-5 index [14] includes ALB, ALP, AST, ALT, and PLT, which may be the reason why the FIB-5 index has predictive value for the occurrence of MACCEs. However, when we carefully analyzed the correlation between the various components of FIB-5 index and MACCEs, we found that AST and ALB were significantly associated with the occurrence of cardiac events (Table 1p < 0.05). This is because AST levels are higher in the hepatic lobular region than ALT, and AST comes from different organs, including the liver, myocardium, skeletal muscle, and red blood cells, while ALT mainly comes from the liver. In addition, the relationship between hypoalbuminemia and poor prognosis of heart failure has also been confirmed [8]. However, in the cardiovascular field, only a few studies have assessed the correlation between the FIB-5 index and the prognosis of patients with heart disease. As reported by Maeda et al. [17], The FIB-5 index predicted the occurrence of cardiac death or HF readmission in patients with AHF, and a low FIB-5 index was significantly associated with poor prognosis. In patients with coronary artery disease undergoing percutaneous coronary intervention, a low FIB-5 index was independently associated with contrast-associated acute kidney injury [29]. This phenomenon that a low FIB-5 index is associated with a high incidence of MACCEs in patients with ADHF was also confirmed in the current study.

### Rationality of the combination of CRP and FIB-5 index

Inflammation has a significant impact on the development of HF [2, 30], and in patients with HFpEF, systemic microvascular inflammation plays a key role in the pathogenesis of structural and functional changes in the myocardium [31]. A recent study showed that in patients with HFrEF [32], sacubitril/valsartan inhibits pro-inflammatory cytokines and slows cardiac remodeling. Patients with HF often show signs of chronic systemic inflammation, such as elevated serum CRP levels [33–35]. Significantly elevated CRP levels on admission may increase the short-term risk of cardiac and noncardiac deaths in patients hospitalized due to AHF [24]. A previous study noted that high CRP levels at discharge from the hospital in patients with ADHF were significantly associated with the risk of death at 1 year [19]. In addition, some researchers showed that hs-CRP is associated with a significantly increased risk of new-onset HF and new-onset diabetes [22].

The FIB-5 index has a high predictive value for outcomes in patients with ADHF because it incorporates a large number of variables related to liver function. Interestingly, CRP also predicts adverse prognosis in patients with HF due to its inflammatory properties. FIB-5 index combined with CRP may be useful for patients with ADHF as a simple, easily accessible, and non-invasive indicator of inflammation and liver function. Previous studies have shown that high CRP levels and low FIB5 are associated with adverse cardiovascular events, while in the current study, we used FIB5 in combination with CRP for the first time for risk stratification in patients with ADHF and demonstrated that this combination could further improve the predictive efficacy of adverse outcomes in patients with ADHF.

In addition, our study further evaluated ADHF outcomes, including all-cause death, non-fatal stroke, non-fatal myocardial infarction and revascularization, malignant arrhythmia, and HF rehospitalization, in patients with established ADHF to suggest the clinical prognosis of ADHF. Considering that ADHF is related to public health and economic burdens, it is crucial to identify and manage patients at high risk for ADHF, especially in economically underdeveloped areas. As mentioned above, cardiohepatic syndrome is an important comorbidity in patients with HF. Although liver biopsy is the gold standard for evaluating liver fibrosis, it is still an invasive and risky procedure. The FIB-5 index and CRP are both simple and easily obtainable laboratory indicators. FIB-5 index combined with CRP can help doctors quickly stratify the risk of ADHF.

### Limitations

Nevertheless, the present study has some limitations. First, it was a retrospective, single-center, observational study with issues, such as missing study subjects, short follow-up, and small sample size. Second, data bias could not be avoided even after adjusting for accepted prognostic factors. Third, as a component of FIB-5 index, AST is significant with MACCEs. However, AST is the least specifically hepatic of the transaminases and could be of muscular or cardiac origin. Fourth, cardio-renal syndrome is a frequent complication of heart failure with a poor prognosis. However, this complication has not been documented and included in fitting models. Finally, although ADHF patients with severe infection were excluded, we could not deny those who are still suspected of infection.

## Conclusion

In conclusion, both FIB-5 index and CRP are easily accessible and non-invasive markers. FIB-5 index combined with CRP can guide clinical physicians in risk stratification of patients with ADHF and thus improve their prognosis.

### Electronic supplementary material

Below is the link to the electronic supplementary material.

Supplementary Material 1

## Data Availability

The information and data of the study population were extracted from the hospital information system. The datasets are not publicly available because the privacy of the participants should be protected. The datasets used and analyzed during the current study are available from the corresponding author upon reasonable request.

## Abbreviations

FIB-5: fibrosis-5
HF: heart failure
AHF: acute heart failure
ADHF: acute decompensated heart failure
CRP: C-reactive protein
HFpEF: HF with preserved ejection fraction
HFrEF: HF with reduced ejection fraction
HFmrEF: HF with mid-range ejection fraction
HR: hazard ratio
ALB: albumin
ALP: alkaline phosphatase
ALT: alanine aminotransferase
PLT: platelet
MACCEs: major adverse cardiac and cerebral events
FIB-4: fibrosis-4
IL-6: interleukin-6
TNF-α: tumor necrosis factor-alpha
IVSTD: interventricular septum thickness
LVPWT: left ventricular posterior wall thickness
LVDd: left ventricular diastolic diameter
AoD: aortic diameter
LAD: left atrial diameter
LVEF: left ventricular ejection fraction
BNP: B-type natriuretic peptide
UA: uric acid
WBC: white blood cell
HDL-C: high-density lipoprotein cholesterol
LDL-C: low-density lipoprotein cholesterol
HbA1c: glycated hemoglobin
FPG: fasting plasma glucose
BUN: blood urea nitrogen
SD: standard deviation
MI: myocardial infarction
IQR: interquartile range
ANOVA: Analysis of variance
ACEI: angiotensin-converting enzyme inhibitor
ARB: angiotensin receptor inhibitors
ARNI: angiotensin receptor neprilysin inhibitor
SGLT2i: sodium-glucose contransporter-2 inhibitor
CI: confidence intervals
NRI: net reclassification improvement
IDI: integrated discrimination improvement
