# Supplementary material for: Prognostic value of fibrosis-5 index combined with C-reactive protein in patients with acute decompensated heart failure
Source: BMC Cardiovasc Disord. 2023 Oct 4;23:492. doi: 10.1186/s12872-023-03530-2 (PMC10552406; doi:10.1186/s12872-023-03530-2)
Supplement: Supplementary file 1 — Supplementary Material 1 [file 12872_2023_3530_MOESM1_ESM.docx]

Supplementary Material

|  | | | | | | | | |
| --- | --- | --- | --- | --- | --- | --- | --- | --- |
|  | **Model 1** | | **Model 2** | | **Model 3** | | |  |
|  | **HR (95% CI)** | **P-value** | **HR (95% CI)** | **P-value** | | **HR (95% CI)** | **P-value** |  |
| FIB-5 index | 0.97(0.96-0.98) | <0.001 | 0.98(0.97-0.99) | <0.001 | | 0.98(0.97-0.99) | 0.001 |  |

Note: FIB-5 as continuous variables

*HR* hazard ratio; *CI* confidence intervals; *FIB-5* fibrosis-5; *CRP* C-reactive protein;

Model 1: adjusted for age, gender, SBP, DBP, heart rate, and BMI.

Model 2: adjusted for Model 1+BNP, hematocrit, UA, WBC, Creatinine, Sodium, Potassium, LDL-C, LAD, and LVEF.

Model 3: adjusted for Model 2+history of hypertension, valvular heart disease, atrial fibrillation, and use of aldosterone antagonist, Beta-Blockers, diuretics, statins, digoxin, ACEI/ARB/ARNI, and SGLT2i.
